# Tertiary Lymphoid Structures in Lupus Nephritis Are Associated With Aging and Chronic Kidney Injury

**DOI:** 10.1016/j.ekir.2026.107001

**Published:** 2026-08-05

**Authors:** Makiko Kondo, Yuki Sato, Shingo Fukuma, Naoya Toriu, Yuichiro Kitai, Keita P. Mori, Takahisa Yoshikawa, Shinya Yamamoto, Naoki Sawa, Yoshifumi Ubara, Motoko Yanagita

**Affiliations:** 1 Department of Nephrology, Graduate School of Medicine, Kyoto University, Kyoto, Japan; 2 Medical Innovation Center TMK Project, Graduate School of Medicine, Kyoto University, Kyoto, Japan; 3 Multiomics Platform, Center for Cancer Immunotherapy and Immunobiology, Graduate School of Medicine, Kyoto University, Kyoto, Japan; 4 Hakubi Center for Advanced Research, Kyoto University, Kyoto, Japan; 5 Department of Epidemiology Infectious Disease Control and Prevention, Graduate School of Biomedical and Health Sciences, Hiroshima University, Hiroshima, Japan; 6 Human Healthcare Science, Graduate School of Medicine, Kyoto University, Kyoto, Japan; 7 Institute for the Advanced Study of Human Biology (WPI-ASHBi), Kyoto University, Kyoto, Japan; 8 Nephrology Center, Toranomon Hospital, Tokyo, Japan

**Keywords:** aging, lupus nephritis, tertiary lymphoid structures, TLSs

## Introduction

Systemic lupus erythematosus (SLE) is a chronic autoimmune disease with diverse clinical manifestations. Lupus nephritis (LN), 1 of the most serious manifestations of SLE, exhibits heterogeneous pathological features.^1^ Although the ISN and/or Renal Pathology Society classification has been expanded to include interstitial lesions in addition to glomerular lesions, the clinical significance of interstitial lesions remains incompletely understood.

Tertiary lymphoid structures (TLSs) are inducible ectopic lymphoid structures that arise in nonlymphoid organs under chronic inflammatory conditions such as cancer, infection, and autoimmunity.^2^^,^S1^,^S2 Although TLS formation has been reported in several forms of chronic kidney disease,S3 their clinicopathological characteristics in LN remain poorly defined.

We previously demonstrated that TLSs preferentially develop in aged kidneys after injury^3^^,^^4^^,^S4 and identified 2 age-associated lymphocyte populations—senescence-associated T cellsS5 and age-associated B cellsS6—that accumulate within kidney TLSs.^5^ Senescence-associated T cells and age-associated B cells interact with one another via the CD153–CD30 signaling, which promotes TLS expansion and kidney dysfunction.^5^ Notably, senescence-associated T cells and age-associated B cells have also been reported to accumulate in kidneys in murine LN.S7–S10 These findings raise the possibility that immune aging contributes to TLS formation in LN. In this study, we investigated the clinicopathological features of kidney TLSs in LN.

A full protocol is provided in the Supplementary Methods.

## Results

### Clinical Characteristics of the Patient Cohort

The clinical and histological characteristics of the patients with LN enrolled in the present study at the time of biopsy are summarized in Supplementary Table S1. The median age of the 205 patients in this study was 37 years (interquartile range [IQR]: 28–49), and 86% of the patients were female. The median serum creatinine level was 0.7 mg/dl (IQR: 0.6–1.0), and the median estimated glomerular filtration rate (eGFR) was 86.4 ml/min per 1.73 m^2^ (IQR: 65.0–100.1). The median proteinuria was 1.4 g/d (IQR: 0.5–3.3). 91 patients (44.4%) received oral prednisolone at > 20 mg/d or any immunosuppressive agent within 1 month before biopsy, whereas 64 patients (31.2%) received no treatment. According to the current ISN and/or Renal Pathology Society classification of LN,S11 3 patients (1.5%) had class I, 29 (14.1%) class II, 59 (28.8%) class III, 78 (38.0%) class IV, and 36 (17.6%) class V. No patients had class VI in this study. The median modified National Institutes of Health activity index and chronicity index (CI)S11 were 4 (IQR: 2–6) and 1 (IQR: 0–3), respectively.

### Characterization of TLSs in LN

**Figure 1:**
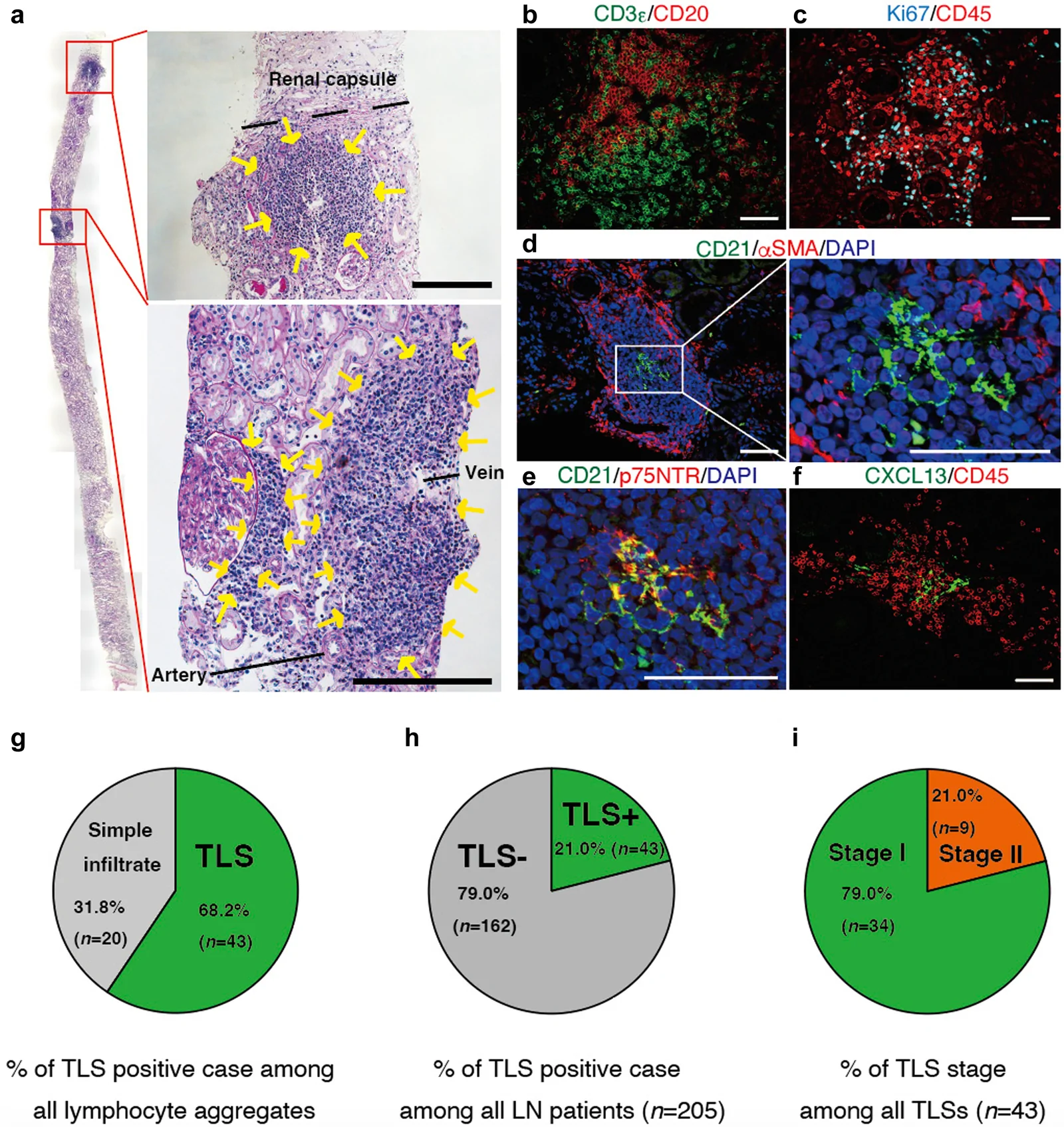
Tertiary lymphoid structures (TLSs) in kidneys from patients with lupus nephritis. Histological analysis of kidneys from patients with lupus nephritis. (a) Periodic acid-Schiff (PAS) staining showed mononuclear cell aggregates just under the kidney capsule, perivascular and periglomerular areas. The right pictures show higher-magnification views of the boxed area in the left picture. Yellow arrows indicate the localization of TLSs. Immunofluorescence staining for (b) CD3ε and CD20; (c) Ki67 and CD45; (d) CD21, a smooth muscle actin (αSMA) and 4′6-diamidino-2-phenylindole (DAPI); (e) CD21, p75NTR, and DAPI; and (f) CXCL13 and CD45. (g–i) Pie charts showing (g) the relative frequency of TLSs among all lymphocyte aggregates, (h) the proportion of TLS-positive cases among all patients with lupus nephritis, and (i) the proportions of stage Ⅰ and stage Ⅱ TLSs among all TLSs. Scale bars: 200 μm in (a) and 50 μm in (b–f).

TLSs were defined as organized T- and B-cell aggregates with proliferative activity, as in our previous study,^6^ and were evaluated as detailed in Supplementary Methods. On Periodic acid-Schiff staining, TLS-like structures (mononuclear cell aggregates) were primarily located in perivascular, subcapsular, and periglomerular regions (Figure 1a), which are sites previously reported to support TLS development in other chronic kidney disease.^6^^,^S12 Immunofluorescence analysis showed that these aggregates consisted mainly of T and B cells and included Ki67-positive proliferating cells, defining these mononuclear cell aggregates as TLSs (Figure 1b and c). Some TLSs contained CD21-positive follicular dendritic cells (Figure 1d), which regulate B cell homeostasis and humoral immune responses.S13 Follicular dendritic cells were also positive for p75 neurotrophin receptor (Figure 1e), a neural crest marker.^3^^,^^6^^,^S14 CXCL13 was expressed in nonhematopoietic cells within TLSs (Figure 1f). Overall, the cellular and molecular features of TLSs in LN kidneys were similar to those of age-dependent TLSs. Among 63 patients with mononuclear cell aggregates, 43 patients (68.2%) met the criteria for TLSs (Figure 1g), corresponding to 21.0% of all patients with LN (Figure 1h). Of these 43 patients, 34 (79.0%) had stage I TLSs and 9 (21.0%) had stage II TLSs (Figure 1i), defined by the absence or presence of CD21^+^ follicular dendritic cells, respectively.^6^

### Association Between Age and TLS Formation in LN

The results of the univariate analysis comparing the clinical characteristics of patients with LN with or without kidney TLSs are summarized in Table 1. Patients with LN with TLSs were significantly older than those without TLSs (median age, 48 vs. 35 years; *P* = 0.0001). At the time of kidney biopsy, serum creatinine levels were higher and eGFR was lower in patients with TLSs than in those without TLSs. In contrast, no significant differences were observed in proteinuria, serum complement components (C3, C4, and CH50), antinuclear antibody titers, anti-double-strand DNA antibody titers, or SLE Disease Activity Index scores. Hypertension was more frequent in the TLS group, whereas the prevalence of diabetes did not differ significantly between the 2 groups. TLSs were also more frequently detected in patients who had received no immunosuppressive treatment before biopsy (treatment category 0). Although the distribution of ISN and/or Renal Pathology Society classes did not differ significantly between patients with or without TLSs, the modified CI was significantly higher in patients with TLSs than in those without TLSs (Table 1).

**Table 1:** Clinical and demographic characteristics of the patients with lupus nephritis with or without kidney TLSs

Parameters	TLS (-)	TLS (+)	*P*-value
Number of patients	162 (79)	43 (21)	
Age (yrs)	35 (27–46)	48 (37–61)	0.0001
Sex (male/female)	18/144	11/32	0.016
Duration (mo)	11 (1–103)	3 (1–104)	0.342
Serum creatinine (mg/dl)	0.7 (0.6–0.9)	0.8 (0.6–1.3)	0.003
eGFR (ml/min per 1.73 m^2^)	90.0 (71.2–100.8)	70.9 (39.5–92.0)	0.0007
Proteinuria (g/24 h)	1.4 (0.5–3.3)	1.8 (0.8–4.2)	0.160
Serum C3 (mg/dl)	49.2 (34.0–72.4)	56.3 (41.0–68.0)	0.353
Serum C4 (mg/dl)	9.2 (4.5–16.7)	12.3 (6.6–19.9)	0.056
Serum CH50 (mg/dl)	25.6 (13.5–36.3)	26 (21.0–36.0)	0.398
Antinuclear antibody titer	1:640 (1:160–1:1280)	1:640 (1:120–1:1280)	0.759
Anti-dsDNA titers (IU/ml)	48.8 (10.0–152.5)	40.0 (10.0–80.0)	0.374
Anti-DNA titers (IU/ml)	43.3 (9.9–118.0)	12.6 (4.2–89.6)	0.091
SLEDAI	12 (9–17)	12 (8–16)	0.077
Diagnostic hypertension	36 (22.2)	19 (44.2)	0.004
Diagnostic diabetes mellitus	8 (4.9)	4 (9.3)	0.279
Immunosuppressive treatment before biopsy			0.143
0: no treatment	45 (27.8)	19 (44.2)	
1: past treatment^a^	8 (4.9)	0 (0)	
2: PSL ≤ 20 mg/d^b^	34 (21.0)	8 (18.6)	
3: PSL > 20 mg/d and/or any of immunosuppressants^b^	75 (46.3)	16 (37.2)	
No treatment (0)	45 (27.8)	19 (44.2)	0.039
Treatment (1,2,3)	117 (72.2)	24 (55.8)	
ISN/RPS class (*n*, %)		0.330	
Class I	2 (1.2)	1 (2.3)	
Class II	27 (16.7)	2 (4.7)	
Class III (+V)	45 (27.8)	14 (32.5)	
Class IV (+V)	59 (36.4)	19 (44.2)	
Class V	29 (17.9)	7 (16.3)	
Class III/IV vs I/II/V (*n*, %)			0.120
Class III/IV	104 (64.2)	33 (76.7)	
Class I/II/V	58 (35.8)	10 (23.3)	
Modified activity index	3 (2–6)	4 (2–6)	0.2233
Glomerular AI	3 (1–5)	3 (1–5)	0.6506
Interstitial AI	1 (0–1)	1 (1–1)	< 0.0001
Modified chronicity index	0.5 (0–2)	3 (2–6)	< 0.0001
Glomerular CI	0 (0–1)	1 (0–2)	< 0.0001
Interstitial CI	0 (0–2)	2 (2–4)	< 0.0001

AI, activity index; Anti-dsDNA, anti-double stranded DNA; CI, chronicity index; eGFR, estimated glomerular filteration rate; RPS, Renal Pathology Society; SLEDAI, SLE Disease Activity Index; PSL, prednisolone; TLS, tertiary lymphoid structures.

Data are presented as the median (interquartile range) or *n* (%).

a Administration of any dose of PSL or immunosuppressants 1 month or more before the biopsy.

b Administration within 1 month before the biopsy.

To determine whether the association between TLS presence and kidney function was independent of age and CI, we performed multivariable logistic regression analyses. In separately adjusted models, the association between TLSs and lower eGFR was attenuated after adjustment for age or CI, but not after adjustment for hypertension (Supplementary Table S2). In contrast, age and CI remained independently associated with TLS presence (age: odds ratio: 1.58 per 10-year increase; 95% confidence interval: 1.14–2.17; *P* = 0.005, CI: odds ratio: 1.56; 95% confidence interval, 1.26–1.94; *P* < 0.001, Supplementary Table S3).

We next examined the age dependence of TLS formation in LN. Stratification of patients into 3 age groups (< 30, 30 ≤ to < 50, and 50 ≤ years) revealed a progressive increase in TLS frequency with age, as follows: 13% (8/62), 16% (16/97), and 41% (19/46), respectively (*P* < 0.001 for trend; Supplementary Figure S1A). Moreover, the odds ratio for TLS presence increased stepwise in patients older than 40 years (Supplementary Figure S1B). To further validate this finding, we reanalyzed data from an independent cohort from a previous study^7^ using our TLS definition and confirmed the age-dependent increase in TLS formation (Supplementary Figure S1C).

## Discussion

In this study, we found that aging is strongly associated with TLS formation in LN in both primary and validation cohorts. We previously showed that TLSs spontaneously develop in the kidney with aging, even in the absence of overt kidney disease, in both mice and humans.^3^^,^^6^^,^S15 Patients with chronic kidney disease aged over 60, but not those under 40, are highly susceptible to kidney TLS development.^3^ In contrast, patients with LN in this study exhibited TLSs as early as 30 years of age, with frequencies increasing with age. Although the reasons for this earlier TLS development are unknown, accelerated immune aging may contribute to earlier TLS development in LN, as suggested in other autoimmune diseases such as rheumatoid arthritis.^8^^,^S16 Indeed, a recent study identified an age-associated CD4^+^ T-cell subpopulation with B cell helper and cytotoxic features in young patients with SLE,S17 suggesting that immune aging may contribute to SLE pathogenesis.

Kidney injury has been implicated in TLS formation in other forms of chronic kidney disease.^6^^,^^9^ In our cohort, the CI was higher in patients with LN with TLS than in those without TLS, suggesting an association with local kidney damage. In contrast, eGFR was not independently associated with TLS presence after adjustment for age and CI. This discrepancy may reflect the distinct dimensions of kidney injury evaluated by these measures. Serum creatinine–based eGFR is insensitive to early or localized kidney injury, whereas CI captures cumulative kidney damage within the biopsied tissue. Given that mean kidney function was preserved in this cohort, this may explain why TLS presence was associated with chronic histologic injury but not associated with eGFR.

This study has limitations. Owing to its retrospective cross-sectional design, causality among aging, chronic kidney injury, and TLS formation could not be determined. Furthermore, this study could not distinguish the contribution of age-related changes from that of LN-specific inflammation or kidney injury to TLS formation.

The findings in this study suggest that aging and chronic kidney injury may promote TLS formation in LN. Further longitudinal and mechanistic studies are needed to clarify the relative contributions of aging and LN-specific inflammatory processes to TLS formation.

## Disclosure

YS and KPM were employed by TMK project, which was a collaborative project between Kyoto University and Mitsubishi Tanabe Pharma. MY receives research grants from Mitsubishi Tanabe Pharma and Boehringer Ingelheim. Other authors report no competing interests.

## Supplementary Material

Supplementary file (PDF) **Supplementary Methods. Supplementary References. Figure S1.** TLS formation in lupus nephritis is age-dependent. **Table S1.** Clinical and histologic characteristics of the patient cohort. **Table S2.** Association between eGFR and TLS presence after separate adjustment for age, chronicity index, or hypertension. **Table S3.** Adjusted odds ratios for TLS presence in a multivariable logistic regression model with simultaneous inclusion of eGFR, age, and chronicity index.

## Footnotes

Supplementary file (PDF)

**Supplementary Methods.**

**Supplementary References.**
**Figure S1.** TLS formation in lupus nephritis is age-dependent. **Table S1.** Clinical and histologic characteristics of the patient cohort. **Table S2.** Association between eGFR and TLS presence after separate adjustment for age, chronicity index, or hypertension. **Table S3.** Adjusted odds ratios for TLS presence in a multivariable logistic regression model with simultaneous inclusion of eGFR, age, and chronicity index.

## References

[1] Hoi A., Igel T., Mok C.C., Arnaud L. (2024). Systemic lupus erythematosus. Lancet.

[2] Sato Y., Silina K., van den Broek M., Hirahara K., Yanagita M. (2023). The roles of tertiary lymphoid structures in chronic diseases. Nat Rev Nephrol.

[3] Sato Y., Mii A., Hamazaki Y. (2016). Heterogeneous fibroblasts underlie age-dependent tertiary lymphoid tissues in the kidney. JCI Insight.

[4] Yoshikawa T., Oguchi A., Toriu N. (2023). Tertiary lymphoid tissues are microenvironments with intensive interactions between immune cells and proinflammatory parenchymal cells in aged kidneys. J Am Soc Nephrol.

[5] Sato Y., Oguchi A., Fukushima Y. (2022). CD153/CD30 signaling promotes age-dependent tertiary lymphoid tissue expansion and kidney injury. J Clin Invest.

[6] Sato Y., Boor P., Fukuma S. (2020). Developmental stages of tertiary lymphoid tissue reflect local injury and inflammation in mouse and human kidneys. Kidney Int.

[7] Steinmetz O.M., Velden J., Kneissler U. (2008). Analysis and classification of B-cell infiltrates in lupus and ANCA-associated nephritis. Kidney Int.

[8] Sato Y., Yanagita M. (2019). Immunology of the ageing kidney. Nat Rev Nephrol.

[9] Lee Y.H., Sato Y., Saito M. (2022). Advanced tertiary lymphoid tissues in protocol biopsies are associated with progressive graft dysfunction in kidney transplant recipients. J Am Soc Nephrol.

