# Supplementary material for: Tertiary Lymphoid Structures in Lupus Nephritis Are Associated With Aging and Chronic Kidney Injury
Source: Kidney Int Rep. 2026 Aug 5;11(10):107001. doi: 10.1016/j.ekir.2026.107001 (PMC13546887; doi:10.1016/j.ekir.2026.107001)
Supplement: Supplementary file 1 [file mmc1.pdf]

Supplementary materials for

Tertiary lymphoid structures in lupus nephritis are associated with aging and chronic kidney injury

Makiko Kondo[1], Yuki Sato[1,2,3,4*], Shingo Fukuma[5,6], Naoya Toriu[1,7], Yuichiro Kitai[1], Keita P Mori[1,2], Takahisa Yoshikawa[1], Shinya Yamamoto[1], Naoki Sawa[8], Yoshifumi Ubara[8] and Motoko Yanagita[1,7*]

[1]Department of Nephrology, [2]Medical Innovation Center TMK Project, [3]Multiomics Platform, Center for Cancer Immunotherapy and Immunobiology, Graduate School of Medicine, Kyoto University, Kyoto, Japan

[4]Hakubi Center for Advanced Research, Kyoto University

[5]Department of Epidemiology Infectious Disease Control and Prevention, Graduate School of Biomedical and Health Sciences, Hiroshima University, Hiroshima, Japan

[6]Human Healthcare Science, Graduate School of Medicine, Kyoto University, Kyoto, Japan

[7]Institute for the Advanced Study of Human Biology (WPI-ASHBi), Kyoto University, Kyoto, Japan

[8]Department of Nephrology, Toranomon Hospital, Tokyo, Japan

[*]Corresponding author:

Motoko Yanagita motoy@kuhp.kyoto-u.ac.jp, Yuki Sato yukisato@kuhp.kyoto-u.ac.jp

The PDF includes

Supplementary Methods

Supplementary Figure S1

Supplementary Table S1-S3

Supplementary References.

Supplementary Methods

Patients

205 patients with biopsy-proven lupus nephritis between 1989 and 2016 were recruited from two Japanese tertiary teaching hospitals, Kyoto University Hospital (Kyoto, Japan) and Toranomon Hospital (Tokyo, Japan). Patients with malignancy, infection or failure to meet the revised American College of Rheumatology classification criteria were excluded. All LN patients fulfill American College of Rheumatology revised classification criteria for SLE[S18-S20] and were aged ≥ 18 years.

Kidney biopsy was performed in accordance with the guidelines of the Japanese Society of Nephrology[S21]. Specifically, kidney biopsy was performed in patients with SLE who developed clinical findings suggestive of kidney involvement, including proteinuria ≥ 0.5 g/day, persistent hematuria (≥ 5 red blood cells per high-power field) with dysmorphic erythrocytes, active urinary sediment or cellular casts, or unexplained elevation in serum creatinine. Kidney biopsy was also considered in patients with impaired kidney function or systemic disease even in the absence of urinary abnormalities to evaluate possible silent lupus nephritis, tubulointerstitial disease, or vasculitis. Pathological diagnoses of lupus nephritis were made independently at each institution by experienced, board-certified nephrologists with expertise in kidney pathology through routine kidney biopsy conferences. In contrast, TLS evaluation was performed as a centralized research assessment at Kyoto University. Consistent with our previous studies, all biopsy specimens from both institutions were reviewed by an experienced, board-certified nephrologist with expertise in kidney pathology and TLS evaluation (M.K.), and equivocal cases were further reviewed with Y.S. to reach a consensus regarding TLS assessment. TLS evaluation was performed in a blinded manner with respect to the clinical information. The study was approved by the Ethics Committees of Kyoto University Graduate School of Medicine and Kyoto University Hospital (approval number: R0254), and Toranomon Hospital (approval number: 1487-B). For this retrospective study using archived samples, the requirement for written informed consent was

waived by the ethics committee, and an opt-out approach was implemented that allowed patients to decline participation.

Immunohistological analysis of TLSs in the kidneys

In the present study, TLSs were defined as organized lymphocyte aggregates with evidence of proliferative activity, as in our previous study[6]. To diagnose kidney TLSs and determine their stages, we performed a two-step analysis. First, cases showing mononuclear cell aggregates in the kidneys on PAS staining were considered candidates for TLS evaluation. Second, using the serial sections from kidney biopsy samples of these cases, we performed immunofluorescence staining for (1) CD3ε and CD20 and (2) Ki67 and CD21, and then determined the presence and stage of TLSs. TLS stage was determined based on the presence of CD21-positive follicular dendritic cells (FDCs) and germinal centers, defined as dense Ki67-positive B cell clusters, within TLSs. TLSs containing neither FDC nor germinal centers were classified as stage I, whereas TLSs containing FDCs but lacking germinal centers were classified as stage II. Patients with at least one TLS in biopsy specimens were included in the TLS-positive group.

Clinical data collection

Medical records were reviewed for demographic characteristics, medical history, medication history, and clinical data. Serum creatinine, eGFR, SLEDAI score, serological markers, and proteinuria data were collected at the time of the biopsy. eGFR was estimated using CKD-EPI 2009 equation with a Japanese coefficient of 0.813[S22].

eGFR ($mL/min/1.73m^2$) = $0.813 \times 141 \times \min(sCr/\kappa, 1)^{\alpha} \times \max(sCr/\kappa, 1)^{-1.209} \times 0.993^{Age} \times 1.018$ (if female), where κ = 0.7 for female and 0.9 for male, and α=-0.329 for female and -0.411 for male. Min (sCr/κ, 1) indicates the minimum of sCr/κ or 1, whereas max (sCr/κ, 1) indictaes the maximum of sCr/κ or 1.

Patients diagnosed with hypertension or receiving antihypertensive medications were defined as

having hypertension. Treatment status at the time of biopsy was categorized into four groups as follows: 0, no medication; 1, treatment with any dose of prednisolone (PSL) or any immunosuppressive agents initiated ≥ 1 month before biopsy; 2, treatment with PSL at ≤ 20mg/day PSL within 1 month before biopsy; 3, treatment with PSL at > 20mg/day of PSL or any immunosuppressive agent within 1 month before biopsy.

Reanalysis of LN data from a previous study

We reanalyzed the association between age and TLS presence using data from a previously published study[7]. In the original classification, grade 1 corresponded to cellular infiltration, whereas grades 2-4 were considered indicative of TLS presence, and their association with age was analyzed.

Statistical analysis

Descriptive statistics are presented as medians and interquartile ranges (IQR). For univariate comparisons between patients with and without TLSs, continuous variables were using the Wilcoxon rank-sum test and the categorical variables using Fisher's exact test. Differences in TLS prevalence across age categories were analyzed using the Cochran-Armitage test for trend. Logistic regression analysis was performed to evaluate factors associated with TLS presence, with TLS presence as the dependent variable. eGFR was analyzed in 10 mL/min/1.73 m^2 increments, age in 10-year increments, chronicity index as a continuous variable, and hypertension as a binary variable. Separate multivariable models were used to assess whether the associations between eGFR and TLS presence was independent of age, chronicity index, or hypertension. Models with simultaneous inclusion of eGFR, age, and chronicity index were also used to assess the odds ratio for TLS presence. To further assess the association between age and TLS presence in Supplementary Figure 1b and 1c, age was modeled as a continuous variable using restricted cubic spline function with four knots, and odds ratios were estimated by logistic regression with 40

years as the reference value. Analysis was performed using Stata version 16 (StataCorp, College Station, TX, USA) and R. Statistical significance was defined as $P < 0.05$.

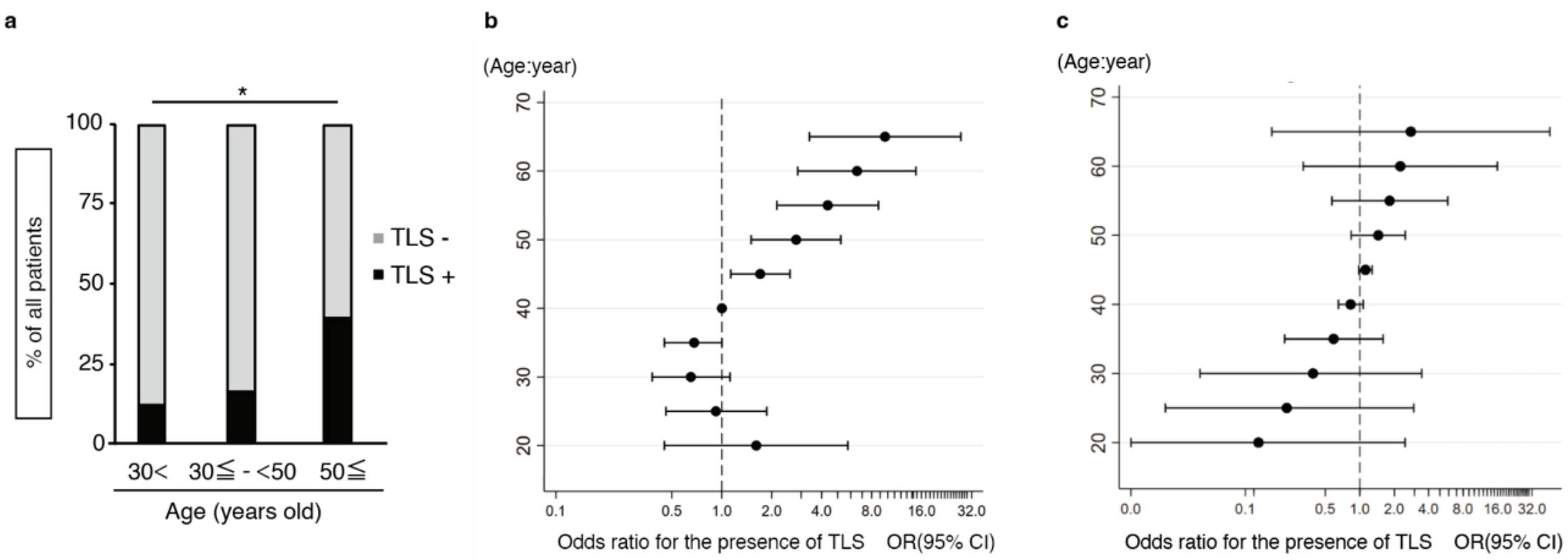

Supplemental Figure S1. TLS formation in lupus nephritis is age-dependent.

(**a**) Frequency of TLSs according to age group (<30, 30≤ - <50, and 50≤ years). * $P < 0.001$ for trend. (**b-c**) Odds ratios with 95% confidence interval (CI) for the association between age and kidney TLS development in patients with lupus nephritis. (**b**) and (**c**) were derived from the current cohort and a previously reported cohort[7], respectively. In (**b**) and (**c**), the reference age was defined as the age of the participant closest to 40 years. Accordingly, the reference age was 40 years in (**b**) and 43 years in (**c**).

Number of patients	205
Age (years)	37 (28-49)
Sex (male/female)	29/176
Duration (months)	7.7 (0.8-102.7)
Serum creatinine (mg/dl)	0.7 (0.6-1.0)
eGFR (ml/min/1.73m^2)	86.4 (65.0-100.1)
Proteinuria (g/24hours)	1.4 (0.5-3.3)
Serum C3 (mg/dl)	50.0 (35.4-71.8)
Serum C4 (mg/dl)	9.9 (5.0-17.0)
Serum CH50 (mg/dl)	25.6 (14.1-36.0)
Antinuclear antibody titer	1:640 (1:160-1:1280)
Anti-dsDNA titers (IU/ml)	45.0 (10.0-120.0)
Anti-DNA titers (IU/ml)	39.0 (9.0-106.9)
SLEDAI	12 (8-16)
Diagnostic hypertension	55 (26.8)
Diagnostic diabetes mellitus	12 (5.9)
Immunosuppressive treatment	
no treatment	64 (31.2)
past treatment[a]	8 (3.9)
PSL≤20mg/day[b]	42 (20.5)
PSL>20mg/day and/or any of immunosuppressants[b]	91 (44.4)
ISN/RPS lupus nephritis class	
Class I	3 (1.5)
Class II	29 (14.1)
Class III (+V)	59 (28.8)
Class IV (+V)	78 (38.0)
Class V	36 (17.6)
Modified NIH activity index	4 (2-6)
Modified NIH chronicity index	1 (0-3)

Supplemental Table S1. Clinical and histologic characteristics of the patient cohort.

Anti-dsDNA, anti-double stranded DNA; SLEDAI, SLE Disease Activity Index; ISN/RPS, International Society of Nephrology/Renal Pathology Society; PSL, prednisolone. Data are presented as the median (interquartile range) or n (%). [a] Administration of any dose of PSL or immunosuppressants one month or more before the biopsy. [b] Administration within one month before the biopsy.

Model	Covariates included in the model	OR for eGFR	95% CI	P Value
1	None	0.81	0.72-0.91	0.001
2	Age, per 10-year increase	0.89	0.77-1.03	0.118
3	Chronicity Index	1.02	0.87-1.21	0.775
4	Hypertension	0.84	0.74-0.95	0.007

Supplemental Table S2. Association between eGFR and TLS presence after separate adjustment for age, chronicity index, or hypertension.

Logistic regression models were used to evaluate the association between eGFR and the TLS presence after separate adjustment for age, chronicity index, or hypertension. Odds ratios for eGFR are shown per 10 ml/min/1.73 m^2 increase. Age was modeled per 10-year increase. Hypertension was included as a binary variable, and Chronicity Index was included as a continuous variable.

Variable	OR	95% CI	*P* Value
eGFR	1.14	0.94-1.39	0.193
Age	1.58	1.14-2.17	0.005
Chronicity Index	1.56	1.26-1.94	<0.001

Supplemental Table S3. Adjusted odds ratios for TLS presence in a multivariable logistic regression model with simultaneous inclusion of eGFR, age, and chronicity index.

Odds ratios for TLS presence is shown per 10 ml/min/1.73 m^2 increase in eGFR, per 10-year increase in age, and per 1-point increase in the chronicity index. Age and chronicity index were included as continuous variables.

Supplementary references

S1. Sato Y, Jain A, Ohtsuki S, et al. Stem-like CD4(+) T cells in perivascular tertiary lymphoid structures sustain autoimmune vasculitis. *Sci Transl Med*. Sep 6 2023;15(712):eadh0380. doi:10.1126/scitranslmed.adh0380

S2. Teillaud JL, Houel A, Panouillot M, Riffard C, Dieu-Nosjean MC. Tertiary lymphoid structures in anticancer immunity. *Nat Rev Cancer*. Sep 2024;24(9):629–646. doi:10.1038/s41568-024-00728-0

S3. Yoshikawa T, Lee YH, Sato Y, Yanagita M. Tertiary lymphoid tissues in kidney diseases: a perspective for the pediatric nephrologist. *Pediatr Nephrol*. May 2023;38(5):1399–1409. doi:10.1007/s00467-022-05770-4

S4. Sato Y, Yanagita M. Immune cells and inflammation in AKI to CKD progression. *Am J Physiol Renal Physiol*. Dec 1 2018;315(6):F1501–F1512. doi:10.1152/ajprenal.00195.2018 doi:10.1681/ASN.0000000000000202

S5. Fukushima Y, Minato N, Hattori M. The impact of senescence-associated T cells on immunosenescence and age-related disorders. *Inflamm Regen*. 2018;38:24. doi:10.1186/s41232-018-0082-9

S6. Cancro MP. Age-Associated B Cells. *Annu Rev Immunol*. Apr 26 2020;38:315–340. doi:10.1146/annurev-immunol-092419-031130

S7. Sakamoto K, Fukushima Y, Ito K, et al. Osteopontin in Spontaneous Germinal Centers Inhibits Apoptotic Cell Engulfment and Promotes Anti-Nuclear Antibody Production in Lupus-Prone Mice. *J Immunol*. Sep 15 2016;197(6):2177–86. doi:10.4049/jimmunol.1600987

S8. Fukushima Y, Sakamoto K, Matsuda M, et al. cis interaction of CD153 with TCR/CD3 is crucial for the pathogenic activation of senescence-associated T cells. *Cell Rep*. Sep 20 2022;40(12):111373. doi:10.1016/j.celrep.2022.111373

S9. Tahir S, Fukushima Y, Sakamoto K, et al. A CD153+CD4+ T follicular cell population with cell-senescence features plays a crucial role in lupus pathogenesis via osteopontin production. *J Immunol*. Jun 15 2015;194(12):5725–35. doi:10.4049/jimmunol.1500319

S10. Rubtsova K, Rubtsov AV, Thurman JM, Mennona JM, Kappler JW, Marrack P. B cells expressing the transcription factor T-bet drive lupus-like autoimmunity. *J Clin Invest*. Apr 3 2017;127(4):1392–1404. doi:10.1172/JCI91250

S11. Bajema IM, Wilhelmus S, Alpers CE, et al. Revision of the International Society of Nephrology/Renal Pathology Society classification for lupus nephritis: clarification of definitions, and modified National Institutes of Health activity and chronicity indices. *Kidney Int*. Apr 2018;93(4):789–796. doi:10.1016/j.kint.2017.11.023

S12. Sato Y, Tamura M, Yanagita M. Tertiary lymphoid tissues: a regional hub for kidney inflammation. *Nephrol Dial Transplant*. Jan 23 2023;38(1):26–33. doi:10.1093/ndt/gfab212

S13. Krimpenfort LT, Degn SE, Heesters BA. The follicular dendritic cell: At the germinal center of autoimmunity? *Cell Rep*. Mar 26 2024;43(3):113869. doi:10.1016/j.celrep.2024.113869

S14. Sato Y, Yanagita M. Functional heterogeneity of resident fibroblasts in the kidney. *Proc Jpn Acad Ser B Phys Biol Sci*. 2019;95(8):468–478. doi:10.2183/pjab.95.033

S15. Toriu N, Sato Y, Kamimura H, et al. Aligning cellular and molecular components in age-dependent tertiary lymphoid tissues of kidney and liver. *PLoS One*. 2025;20(2):e0311193. doi:10.1371/journal.pone.0311193

S16. Zhao TV, Sato Y, Goronzy JJ, Weyand CM. T-Cell Aging-Associated Phenotypes in Autoimmune Disease. *Front Aging*. 2022;3:867950. doi:10.3389/fragi.2022.867950

S17. Goto M, Takahashi H, Yoshida R, et al. Age-associated CD4(+) T cells with B cell-promoting functions are regulated by ZEB2 in autoimmunity. *Sci Immunol*. Mar 29 2024;9(93):eadk1643. doi:10.1126/sciimmunol.adk1643

S18. Tan EM, Cohen AS, Fries JF, et al. The 1982 revised criteria for the classification of systemic lupus erythematosus. *Arthritis Rheum*. Nov 1982;25(11):1271–7. doi:10.1002/art.1780251101

S19. Hochberg MC. Updating the American College of Rheumatology revised criteria for the classification of systemic lupus erythematosus. *Arthritis Rheum*. Sep 1997;40(9):1725. doi:10.1002/art.1780400928

S20. Petri M, Orbai AM, Alarcon GS, et al. Derivation and validation of the Systemic Lupus International Collaborating Clinics classification criteria for systemic lupus erythematosus. *Arthritis Rheum*. Aug 2012;64(8):2677–86. doi:10.1002/art.34473

S21. Ubara Y, Kawaguchi T, Nagasawa T, et al. Kidney biopsy guidebook 2020 in Japan. *Clinical and Experimental Nephrology.* 2021;25:325-364. doi:10.1007/s10157-020-01986-6.

S22. Horio M, Imai E, Yasuda Y, Watanabe T, Matsuo S. Modification of the CKD epidemiology collaboration (CKD-EPI) equation for Japanese: accuracy and use for population estimates. *Am J Kidney Dis*. Jul 2010;56(1):32–8. doi:10.1053/j.ajkd.2010.02.344
